# What Brings Joy to LTC Providers: View From the Editors’ Desk

**DOI:** 10.1093/geroni/igaf122.633

**Published:** 2025-12-31

**Authors:** Elizabeth Galik, Tess Bird

**Affiliations:** University of Maryland School of Nursing, Baltimore, Maryland, United States; Elsevier Publishing, Philadelphia, Pennsylvania, United States

## Abstract

Recruiting and retaining competent and caring post-acute and long-term care (PALTC) clinicians is critical for quality and safe patient care. Identification and promotion of activities that foster joy in work for PALTC clinicians is protective against burnout which has increased in severity and prevalence since the COVID-19 pandemic. Qualitative methods were used to analyze ten essays solicited by and submitted to Caring for the Ages, a publication of the Post-Acute and Long-Term Care Medical Association (PALTmed). Authors were asked to write in the first person about a work experience in PALTC that made them feel good and bring joy to their work as clinicians. Essays were analyzed using content analysis. Three themes were identified: (1) Building relationships through unique life stories; (2) Collaborating as a team to provide a sense of belonging and meaningful engagement among residents and staff; and (3) Fostering shared decision making that supports person centered care at the end of life. Disciplines that were represented included medicine, nursing and social work. Despite challenges of increasing workload and staffing shortfalls, clinicians may be able to increase joyful experiences in their clinical practice in PALTC through engagement in narrative medicine, supporting and celebrating the dedication of direct care staff, and recognizing the importance of shared medical decision making, particularly within a palliative care context.

